# Supplementary material for: Interpretable deep learning for chromatin-informed inference of transcriptional programs driven by somatic alterations across cancers
Source: Nucleic Acids Res. 2022 Oct 16;50(19):10869–81. doi: 10.1093/nar/gkac881 (PMC9638905; doi:10.1093/nar/gkac881)
Supplement: gkac881_Supplemental_Files [file gkac881_supplemental_files.zip › Supplementary_CITRUS-NAR-revised.pdf]

Supplementary material:

Interpretable deep learning for chromatin-informed inference of transcriptional programs driven by somatic alterations across cancers

Yifeng Tao[1+], Xiaojun Ma[2,3+], Drake Palmer[3], Russell Schwartz[1,4], Xinghua Lu[2,5], Hatice Ulku Osmanbeyoglu[2,3,6,7*]

[1]Computational Biology Department, School of Computer Science, Carnegie Mellon University, Pittsburgh, PA, USA
[2]Department of Biomedical Informatics, School of Medicine, University of Pittsburgh, Pittsburgh, PA, USA
[3]UPMC Hillman Cancer Center, University of Pittsburgh, Pittsburgh, PA, USA
[4]Department of Biological Sciences, Carnegie Mellon University, Pittsburgh, PA, USA
[5]Department of Pharmaceutical Science, School of Medicine, University of Pittsburgh, Pittsburgh, PA, USA
[6]Department of Bioengineering, School of Engineering, University of Pittsburgh, Pittsburgh, PA, USA
[7]Department of Biostatistics, School of Public Health, University of Pittsburgh, Pittsburgh, PA, USA

[+]These authors contributed equally: Y.T., X.M.

[*] Correspondence to: Hatice Ulku Osmanbeyoglu (osmanbeyogluhu@pitt.edu)
ORCID ID: 0000-0002-4972-4347

Supplementary Methods
Details of multi-head self-attention mechanism

We implemented the multi-head self-attention mechanism through a sub neural network. We calculated the tumor embedding $\boldsymbol{e}_t$ of cancer patient t with cancer type s and m somatic alterations $\{g_u\}_{u=1}^{m}$ through the weighted sum of the corresponding gene embeddings $\{\boldsymbol{e}_u\}_{u=1}^{m}$ and cancer type embedding $\boldsymbol{e}_s$ of the tumor:

$$\boldsymbol{e}_t = \boldsymbol{e}_s + \alpha_1\boldsymbol{e}_1 + \alpha_2\boldsymbol{e}_2 + \alpha_3\boldsymbol{e}_3 + \cdots + \alpha_m\boldsymbol{e}_m.$$

The weights $\{\alpha_u\}_{u=1}^{m}$ are determined by the self-attention mechanism. The self-attention mechanism outputs the attention weights by taking as input the gene embeddings $\{\boldsymbol{e}_u\}_{u=1}^{m}$:

$$\alpha_1, \alpha_2, \ldots, \alpha_m = SelfAttention(\boldsymbol{e}_1, \boldsymbol{e}_2, \ldots, \boldsymbol{e}_m).$$

To be more specific, we first calculated the unnormalized attention weights of somatic alterations $\{\beta_{u,j}\}_{u=1}^{m}$ using the j-th head through:

$$\beta_{u,j} = \boldsymbol{\theta}_j^{\intercal} tanh(W_0\boldsymbol{e}_u), \qquad u = 1,2, \ldots, m.$$

Then the attention weights are normalized through a softmax activation:

$$\alpha_{1,j}, \alpha_{2,j}, \ldots, \alpha_{m,j} = softmax(\beta_{1,j}, \beta_{2,j}, \ldots, \beta_{m,j}).$$

In the case with h attention heads, we have different attention head parameters $\{\boldsymbol{\theta}_j\}_{j=1}^{h}$, and the final attention weights is the sum of attention weights from each head:

$$\alpha_u = \alpha_{u,1} + \alpha_{u,2} + \cdots + \alpha_{u,h}, \qquad u = 1,2, \ldots, m.$$

Supplementary Figures

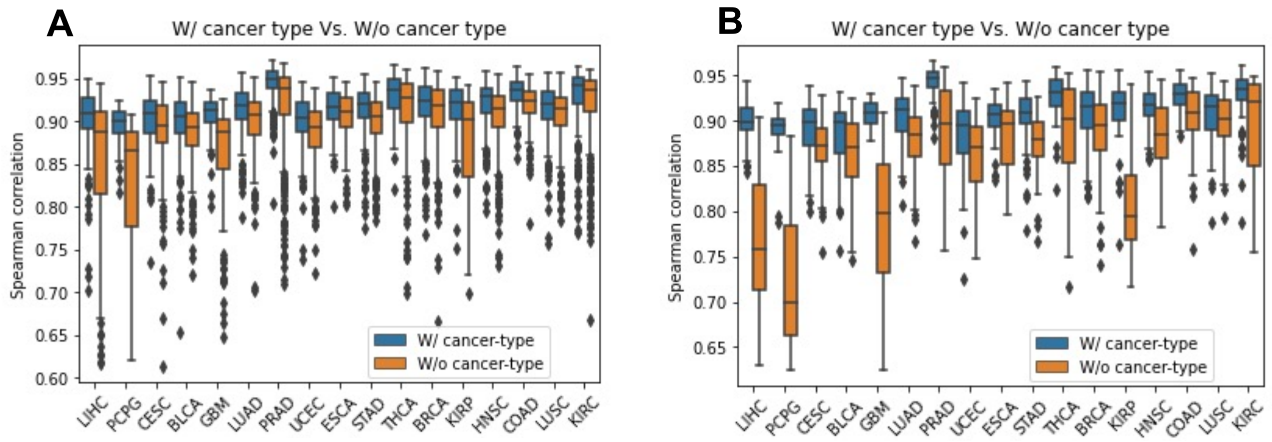

Supplementary Fig. 1: Comparison of models built with and without cancer type embedding. (A) The correlation between predicted and actual gene expression on all data except for the hold-out test set. Those samples were involved in the training process, either appearing in the training set or validation set. **(B)** The correlation between predicted and actual gene expression on hold-out test-set. This data had never appeared in the training process.

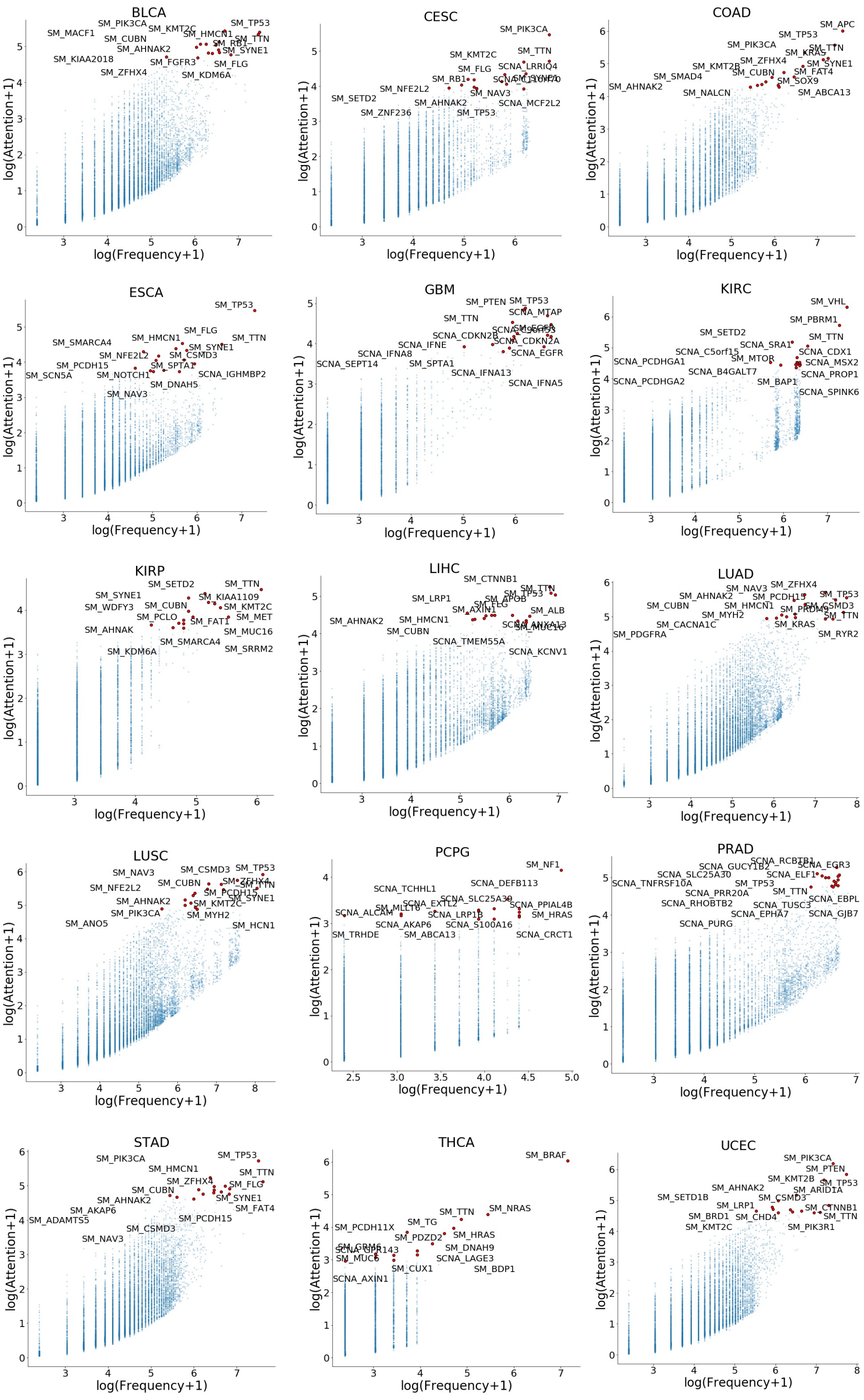

Supplementary Fig. 2: CITRUS-inferred attention weights of genes and mutation frequencies in the individual 15 cancer types.

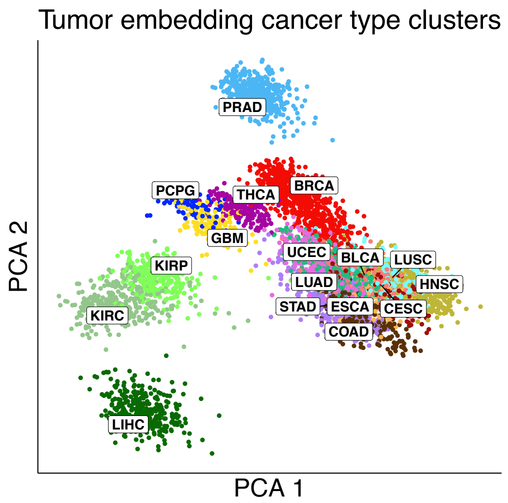

Supplementary Fig. 3: The PCA plot of the CITRUS-inferred tumor embeddings.

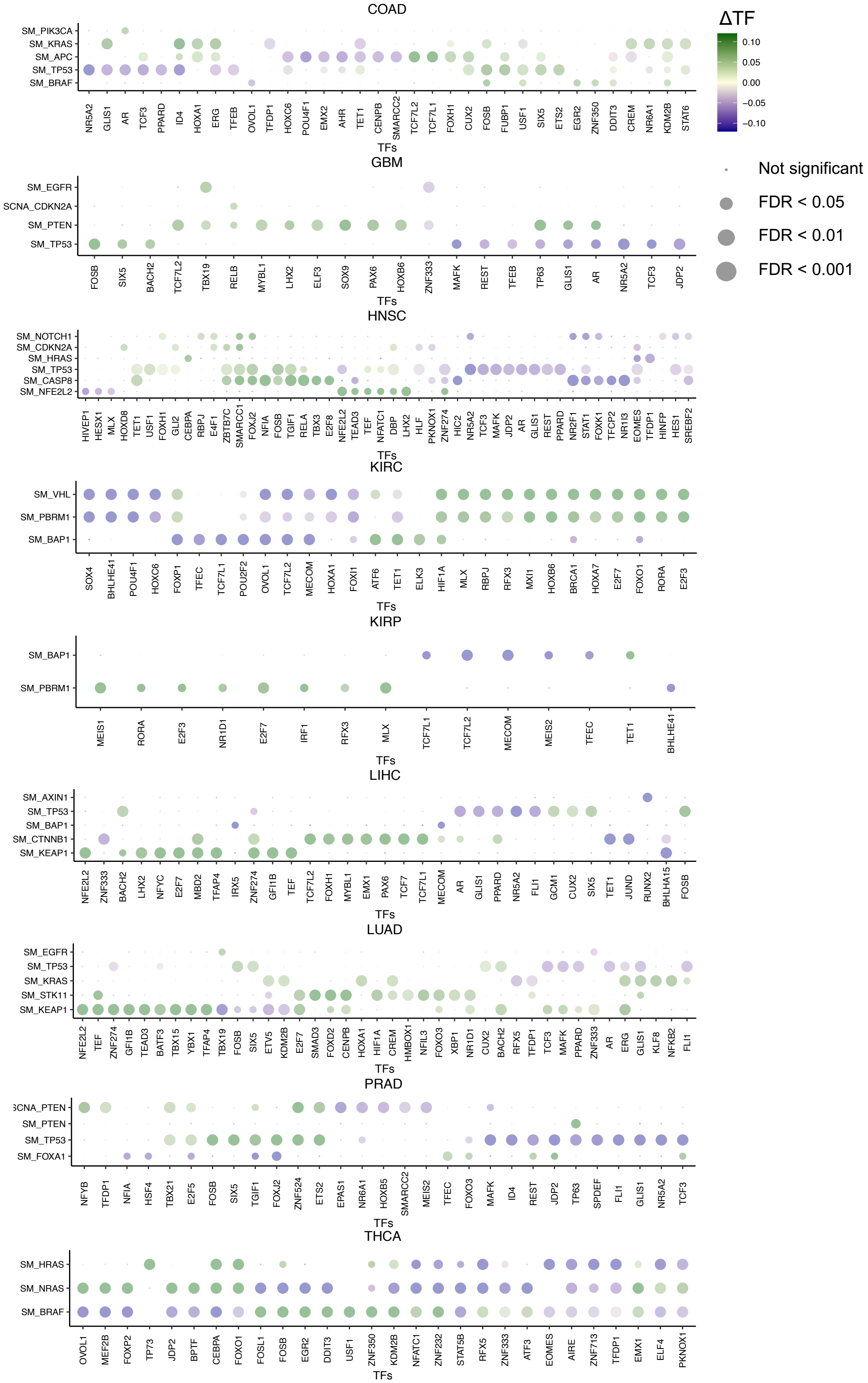

Supplementary Fig. 4: Inferred different TF activities perturbed by somatic alterations in different cancer types. Dotplots show mean TF activity and dot size indicates -$\log_{10}$(FDR).

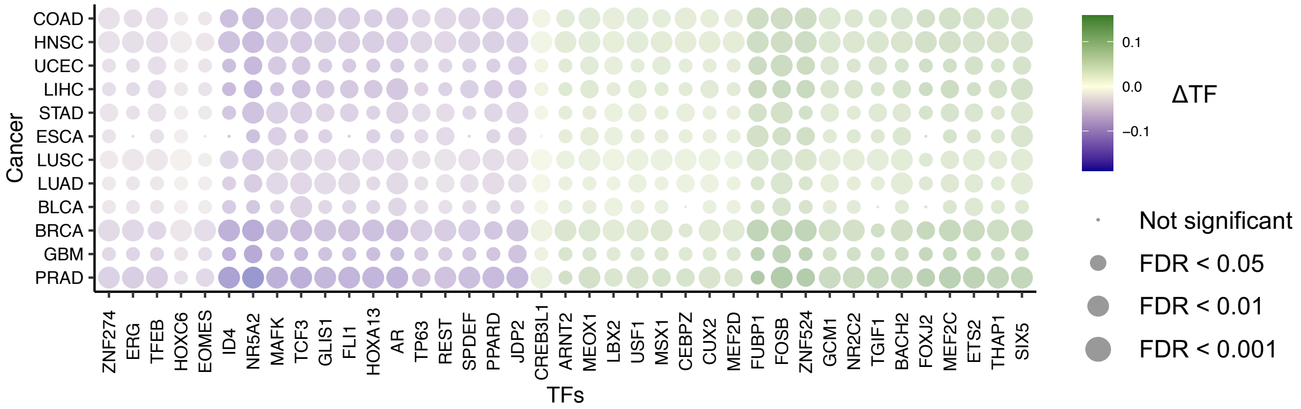

Supplementary Fig. 5: Mutations in TP53 dysregulate similar TFs across cancer types. The dotplot show mean TF activity and dot size indicates -$\log_{10}$(FDR).

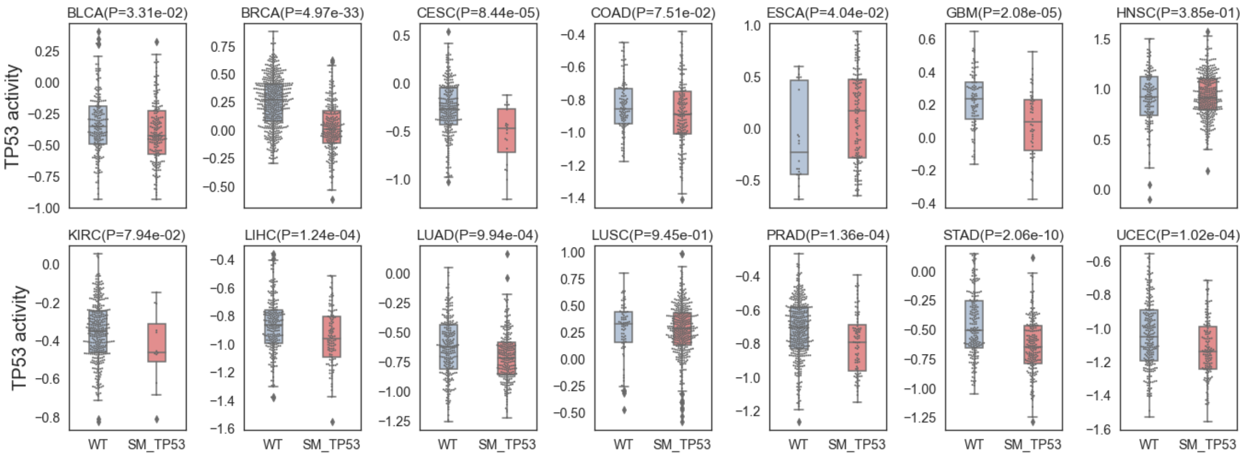

Supplementary Fig. 6: Inferred TP53 activity as a function of mutational status. Tumors with mutant *TP53* have significantly lower TP53 activity than WT tumors ($P < 0.05$, t-test) for most cancer types.

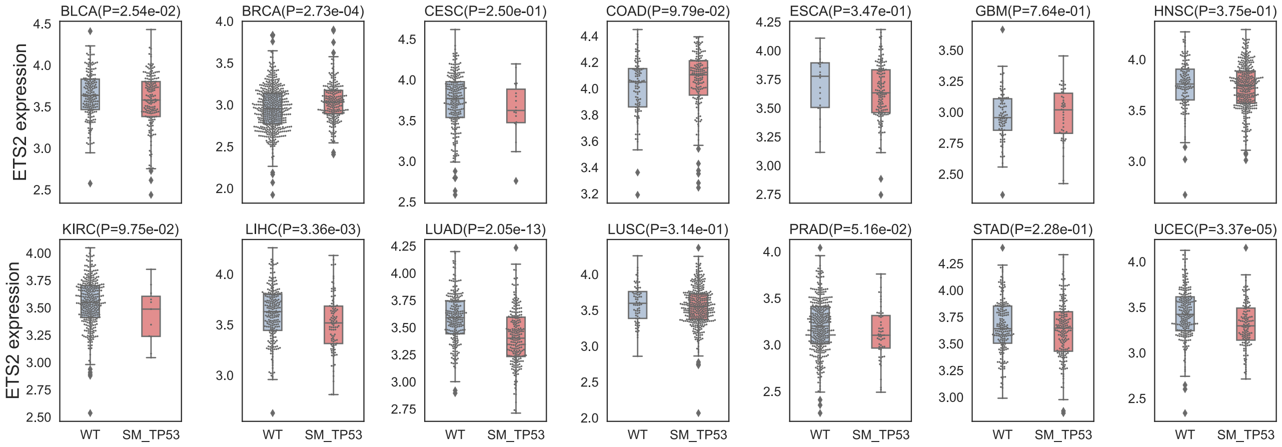

Supplementary Fig. 7: The ETS2 expression levels in wild type and *TP53* mutant TCGA samples per cancer type. Box edges represent the upper and lower quantile with median value shown as bold line in the middle of the box. Whiskers represent 1.5 times the quantile.

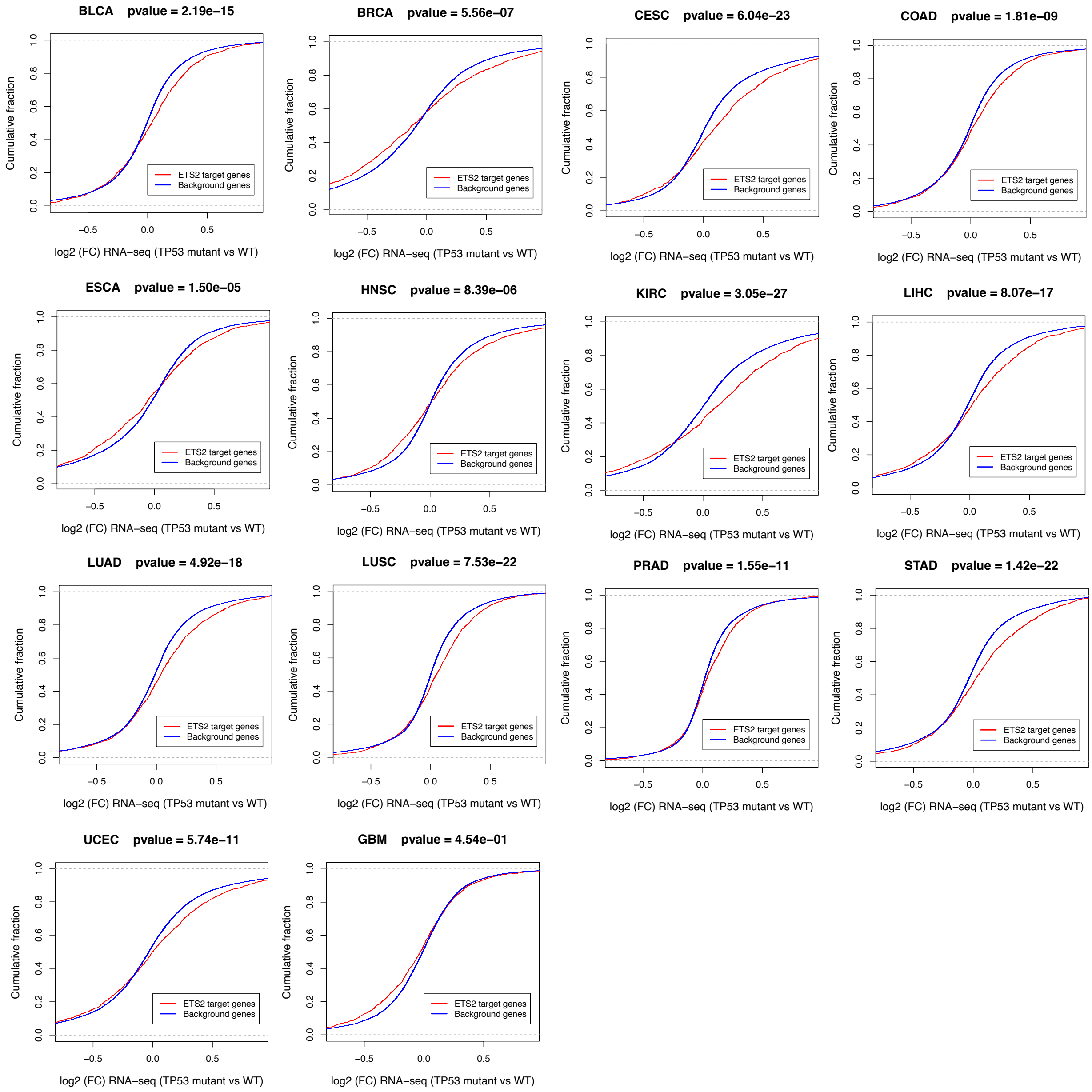

Supplementary Fig. 8: Targets of ETS2 showed significant upregulation in TP53 mutant tumors (p-value < 1e−5, Kolmogorov-Smirnov test) compared to background genes.

Supplementary Tables

Supplementary Table 1: Number of samples in training + validation (for cross-validation) and hold-out set

Cancer Type	training & validation set	hold-out set
BLCA	297	75
BRCA	576	144
CESC	214	53
COAD	217	54
ESCA	136	34
GBM	114	29
HNSC	380	95
KIRC	286	71
KIRP	218	55
LIHC	270	67
LUAD	374	94
LUSC	349	87
PCPG	87	22
PRAD	359	90
STAD	303	76
THCA	173	43
UCEC	289	72

Supplementary Table 2: Performance of the CITRUS models build with actual gene expression compared to randomized gene expression from same sample type and across cancer types. Mean±standard deviation Spearman correlation and mean squared error (MSE) between predicted and actual gene expression changes on held-out samples using the CITRUS model.

Cancer Type	Prediction performance with Spearman correlation			Prediction performance with Mean squared error		
	Actual gene expression	Random sample within same cancer type	Random sample across all samples	Actual gene expression	Random sample within same cancer type	Random sample across all samples
BLCA	0.900±0.03	0.873±0.039	0.722±0.07	0.192±0.052	0.239±0.066	0.560±0.142
BRCA	0.919±0.027	0.902±0.035	0.740±0.071	0.156±0.046	0.185±0.062	0.525±0.144
CESC	0.905±0.029	0.884±0.037	0.718±0.089	0.183±0.049	0.221±0.064	0.568±0.18
COAD	0.933±0.019	0.927±0.022	0.727±0.078	0.134±0.033	0.146±0.039	0.554±0.156
ESCA	0.911±0.029	0.874±0.036	0.723±0.084	0.175±0.051	0.240±0.063	0.564±0.172
GBM	0.906±0.022	0.903±0.024	0.622±0.069	0.181±0.034	0.185±0.038	0.767±0.145
HNSC	0.922±0.025	0.909±0.03	0.723±0.088	0.151±0.044	0.175±0.052	0.561±0.178
KIRC	0.929±0.035	0.921±0.041	0.697±0.069	0.139±0.061	0.154±0.073	0.610±0.139
KIRP	0.913±0.029	0.903±0.037	0.684±0.068	0.169±0.050	0.186±0.064	0.636±0.136
LIHC	0.906±0.028	0.895±0.038	0.628±0.079	0.181±0.047	0.200±0.065	0.775±0.168
LUAD	0.914±0.025	0.899±0.029	0.735±0.067	0.167±0.042	0.191±0.05	0.540±0.138
LUSC	0.916±0.026	0.900±0.031	0.732±0.078	0.162±0.045	0.191±0.054	0.542±0.16
PCPG	0.896±0.021	0.895±0.022	0.584±0.079	0.202±0.034	0.203±0.036	0.832±0.16
PRAD	0.945±0.019	0.941±0.022	0.721±0.074	0.111±0.035	0.118±0.039	0.565±0.152
STAD	0.914±0.026	0.895±0.034	0.736±0.074	0.169±0.046	0.203±0.057	0.540±0.15
THCA	0.930±0.027	0.924±0.03	0.712±0.061	0.138±0.048	0.148±0.053	0.580±0.127
UCEC	0.900±0.03	0.887±0.032	0.722±0.064	0.194±0.05	0.217±0.054	0.564±0.128

Supplementary Table 3: Summary of P-values for driver enrichment and attention weight analysis for top frequently mutated genes

	25%	50%	75%
Driver enrichment *P*-value for high attention driver	**0.025**	**0.014**	**0.001**
Driver enrichment *P*-value for low attention driver	1.000	0.998	1.000

Supplementary Table 4: Comparison of PIK3CA mutation and TF activity & TF mRNA association analysis with SILAC-based phosphoproteomic analysis using isogenic knock-in breast cell lines harboring mutations of *PIK3CA*

	**Based on CITRUS TF activity**		**Based on TF mRNA levels**	
**TF**	***P***	***Adj P***	***P***	***Adj P***
ARID3A	1.02E-01	1.25E-01	8.08E-01	8.50E-01
CEBPB	**8.83E-04**	**1.40E-03**	**1.24E-03**	**3.95E-03**
DNMT1	9.86E-01	9.86E-01	**7.63E-04**	**2.53E-03**
ELK3	**6.96E-04**	**1.14E-03**	**1.44E-02**	**3.62E-02**
ENO1	9.29E-01	9.37E-01	**3.71E-04**	**1.33E-03**
ETV6	**9.63E-03**	**1.39E-02**	3.39E-01	4.56E-01
FOSL1	**9.34E-03**	**1.35E-02**	3.07E-02	7.03E-02
FOXC1	**1.09E-08**	**3.00E-08**	**1.73E-09**	**2.39E-08**
FOXO3	**6.53E-06**	**1.29E-05**	**1.79E-06**	**1.11E-05**
FOXP1	**4.63E-05**	**8.41E-05**	**7.22E-14**	**5.51E-12**
HMGA2	**6.93E-11**	**2.65E-10**	7.33E-01	7.95E-01
JUND	**9.36E-31**	**1.07E-28**	5.78E-01	6.69E-01
MAX	**1.12E-10**	**4.20E-10**	**1.15E-05**	**5.60E-05**
NFATC1	**5.88E-11**	**2.28E-10**	5.96E-01	6.82E-01
RUNX1	4.94E-02	6.42E-02	**3.33E-07**	**2.68E-06**
STAT3	**1.03E-08**	**2.89E-08**	**2.76E-04**	**1.02E-03**
TCF3	**5.72E-18**	**7.28E-17**	8.00E-01	8.49E-01
TFEB	**2.40E-23**	**9.16E-22**	8.46E-01	8.77E-01
TP63	**2.24E-08**	**6.12E-08**	**7.73E-05**	**3.16E-04**
YBX1	**5.92E-08**	**1.49E-07**	**1.78E-09**	**2.39E-08**

Supplementary Table 5: Comparison of PIK3CA mutation and TF activity & TF mRNA levels association analysis with protein microarray-based AKT1 kinase assay

	**Based on CITRUS TF activity**		**Based on TF mRNA levels**	
**TF**	***P***	***Adj P***	***P***	***Adj P***
ATF6	**5.11E-08**	**1.30E-07**	2.15E-01	3.37E-01
BCL11A	**3.92E-06**	**8.16E-06**	**6.32E-06**	**3.37E-05**
MEF2C	**9.64E-20**	**1.84E-18**	2.89E-01	4.15E-01
NR1D1	**2.28E-08**	**6.15E-08**	3.88E-01	5.08E-01
SMAD3	**9.19E-17**	**8.77E-16**	2.55E-01	3.78E-01
SOX9	1.04E-01	1.27E-01	5.20E-01	6.24E-01
STAT6	**1.98E-12**	**9.42E-12**	2.62E-02	6.06E-02
